# Locally Phase-Engineered MoTe_2_ for Near-Infrared Photodetectors

**DOI:** 10.1021/acsphotonics.4c00896

**Published:** 2024-09-16

**Authors:** Jan Hidding, Cédric A. Cordero-Silis, Daniel Vaquero, Konstantinos P. Rompotis, Jorge Quereda, Marcos H. D. Guimarães

**Affiliations:** † Zernike Institute for Advanced Materials, University of Groningen, 9747 AG Groningen, The Netherlands; ‡ Nanotechnology Group, USAL—Nanolab, Universidad de Salamanca, E-37008 Salamanca, Spain; § Departamento de Física de Materiales, GISC, Universidad Complutense de Madrid, E-28040 Madrid, Spain

**Keywords:** scanning photocurrent, transition-metal dichalcogenides, crystal phase-engineering

## Abstract

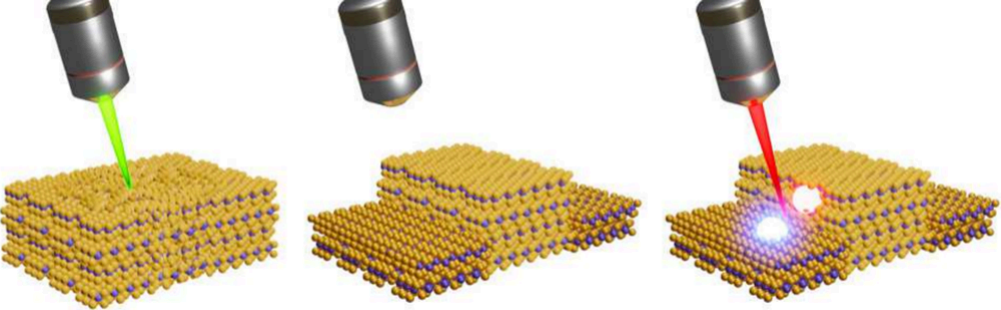

Transition-metal dichalcogenides (TMDs) are ideal systems for two-dimensional (2D) optoelectronic applications owing to their strong light-matter interaction and various band gap energies. New techniques to modify the crystallographic phase of TMDs have recently been discovered, allowing the creation of lateral heterostructures and the design of all-2D circuitry. Thus, far, the potential benefits of phase-engineered TMD devices for optoelectronic applications are still largely unexplored. The dominant mechanisms involved in photocurrent generation in these systems remain unclear, hindering further development of new all-2D optoelectronic devices. Here, we fabricate locally phase-engineered MoTe_2_ optoelectronic devices, creating a metal (1T′) semiconductor (2H) lateral junction and unveil the main mechanisms at play for photocurrent generation. We find that the photocurrent originates from the 1T′–2H junction, with a maximum at the 2H MoTe_2_ side of the junction. This observation, together with the nonlinear IV-curve, indicates that the photovoltaic effect plays a major role in the photon-to-charge current conversion in these systems. Additionally, the 1T′–2H MoTe_2_ heterojunction device exhibits a fast optoelectronic response over a wavelength range of 700–1100 nm, with a rise and fall times of 113 and 110 μs, respectively, 2 orders of magnitude faster when compared to a directly contacted 2H MoTe_2_ device. These results show the potential of local phase-engineering for all-2D optoelectronic circuitry.

## Introduction

Historically, low-dimensional systems have been proposed, fabricated, and studied for enhanced photodetection^1,2^ and potential on-chip photonic applications.^3−5^ Particularly, two-dimensional (2D) materials beyond graphene have shown high interest due to their strong light-matter interaction and compatibility with silicon photonic new generation devices.^6−8^ From the large family of 2D crystals, the group of materials called the transition-metal dichalcogenides (TMDs) have gained much attention in the last two decades due to their versatility, mechanical strength, atomically flat interfaces, and strong absorption at the monolayer limit, making them promising candidates for future (opto)electronic and (opto)spintronic applications.^9^ The most commonly studied crystal structure of the TMD family is the hexagonal (2H) phase, for which most TMDs are semiconducting and possess a thickness-dependent band gap.^10,11^ Apart from the 2H phase, however, TMDs can present a multitude of different crystallographic phases, such as the semiconducting 3R phase, or the semimetallic 1T, 1T′, and 1*T*_d_ phases, which possess different symmetries and (opto)electronic properties. To benefit from these different properties in a single device, researchers have recently focused on gaining control of the crystallographic phase of TMDs, allowing them to transform the phase of single TMD crystals at will.^12,13^ This new and emerging field is now termed the field of phase-engineering and opens the door to creating on-chip 2D circuitry with 2D metals and semiconductors.

In the literature, multiple methods are used to induce a 2H to 1T′ phase transformation for different TMDs, such as crystal deformation,^14−17^ electrostatic doping,^18^ chemical doping,^19,20^ laser heating,^21^ and so forth. In particular, MoTe_2_ gained much attention as the energy barrier between the 2H and 1T′ phase is the smallest (∼40 meV).^22^ This low-energy barrier allows, for moderate laser power, to induce a phase change in MoTe_2_. It was demonstrated, using X-ray and electron microscopy techniques, that the 2H phase evolves into a 1T′ by thermodynamically driven processes, changing the system into a 2D lateral junction.^21,23,24^ It was shown electrically that the Schottky barrier, present when directly contacting the 2H TMD with metallic contacts, is significantly reduced when contacting a 2H TMD via a phase-transformed 1T′ region,^19,21,25,26^ with these two-dimensional lateral junctions, approaching the quantum limit for the contact resistance.^27^ The possibility of fabricating high-quality contacts for a 2D semiconductor is essential to increase the speed of optoelectronic devices based on these materials.

Apart from electrical characterization, only a few reports explored the benefits of local phase engineering on the optoelectronic performance of TMD devices.^28−30^ Lin et al. report an increased responsivity for 2H MoTe_2_ devices using 1T′ interlayer contacts.^29^ However, no scanning photocurrent measurements are performed, which makes it difficult to disentangle the possible microscopic mechanism involved in the photocurrent generation to either the photovoltaic effect (PVE), due to the build in electric field at the Schottky barriers, or the photothermal effect (PTE), due to the different Seebeck coefficients of the 2H and 1T′ regions.^9,31,32^

Here, we perform scanning photocurrent measurements on 1T′–2H MoTe_2_ heterojunction devices, which allow us to spatially resolve the areas involved in the photocurrent generation, giving insights into the underlying mechanisms involved. First, we phase-transform the sides of an exfoliated 2H MoTe_2_ crystal to a 1T′ phase using local heating by laser irradiation, which allows us to contact 2H MoTe_2_ via the semimetallic 1T′ regions. We find a clear nonlinear behavior for the 1T′–contacted 2H region, indicative of a Schottky barrier between the 1T′ and 2H regions. Additionally, using the scanning photocurrent measurements, we clearly observe that the photocurrents are generated at the 1T′–2H junction rather than at the Ti/Au electrodes or the 1T′ region. More specifically, we find that the peak of the photocurrent is generated at the 2H side of the junction, which suggests that the observed photocurrents in the 1T′–2H junction can be attributed to the PVE rather than the PTE. Lastly, we characterize the optoelectronic performance of the MoTe_2_ photodetector by performing time-resolved and laser power-dependent photocurrent measurements. We find fast rise and fall times of 113 and 110 μs, respectively, over a broad spectral range of 700–1100 nm. By comparing our 1T′–2H MoTe_2_ photodetector to a 2H MoTe_2_ diode where the electrodes are directly deposited on the 2H MoTe_2_ crystal, we are able to show that the temporal response of 1T′–contacted 2H MoTe_2_ is 2 orders of magnitude faster. This indicates that phase-engineering can be considered another tool for improving the performance of TMD-based optoelectronic devices.

## Results and Discussion

### Raman Spectroscopy

**Figure 1:**
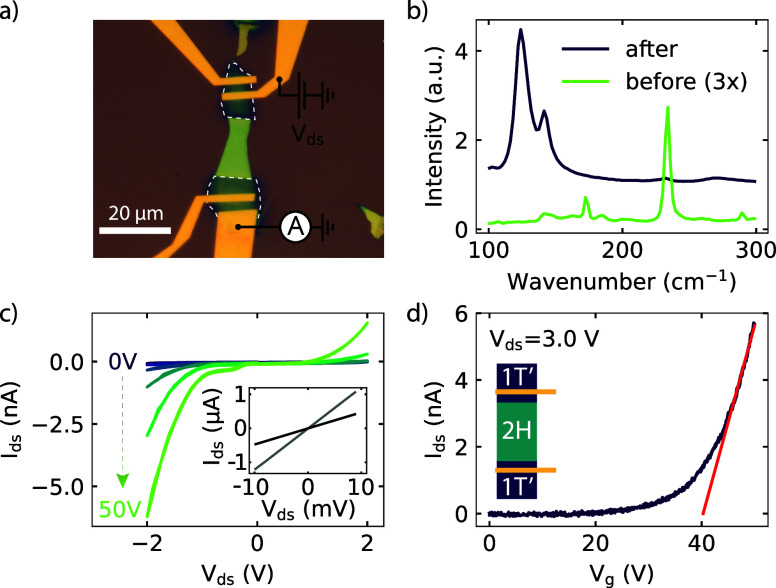
(a) Optical micrograph of a phase-changed MoTe_2_ device, where the phase-changed regions are outlined with the white dashed line, while the bright green part is the unaltered 2H MoTe_2_ region. (b) Raman spectra obtained before (green) and after (purple) the phase transformation, which clearly indicate a successful phase transformation. The spectra before the phase change is multiplied by 3 for clarity. (c) *I*_ds_–*V*_ds_ measurements, as indicated in (a), with *V*_g_ ranging from 0 to 50 V, taken at 78 K. The nonlinear IV characteristics show the Schottky behavior. The IV measurement for the two 1T′ regions are depicted in the inset, which clearly show Ohmic behavior. (d) Transfer curve measured with a *V*_ds_ of 3 V, taken at 78 K, shows a clear *n*-type behavior.

The device used to perform the optoelectronic measurements is depicted in Figure 1a. The green region is the untreated 2H MoTe_2_, while the dark green regions, indicated by the dashed white line, were irradiated with a laser to induce the phase transformation from 2H to 1T′ (details of the device fabrication can be found in the Methods section). To confirm the phase transformation, we performed Raman spectroscopy measurements, as depicted in Figure 1b. Before laser irradiation, we observe the in-plane *E*_2g_ mode at 235 cm^–1^ and an out-of-plane *A*_g_ mode near 174 cm^–1^, indicative of the 2H MoTe_2_ phase. After laser irradiation, these peaks are strongly suppressed, and we observe two new peaks at 124 and 138 cm^–1^, corresponding to the *A*_g_ mode of 1T′ MoTe_2_. This significant change in the Raman spectrum indicates the successful phase transformation of the irradiated regions. To quantify the degree of phase change in our devices, we calculated the spectral weight of each peak in the phase-changed device (see Supporting Information Figure S5 and Table S1), finding values of 0.01 for the peak at ∼232 cm^–1^ and of 2.14 for the peak at ∼124 cm^–1^, corresponding to the 2H and the 1T′ phases, respectively. Our results are in agreement with previous reports, which extensively characterized the laser-induced phase change with several techniques additional to Raman spectroscopy, including X-ray diffraction and electron microscopy.^21,23,24^

### Electrical Characterization

After fabricating electrical contacts to the phase-changed region, we electrically characterized the device in a cryostat at 78 K. We swept the drain-source voltage (*V*_ds_) and measured the drain-source current (*I*_ds_) for the different regions (both 1T′ and the 1T′–2H junction). In Figure 1c, the 2-probe *I*_ds_–*V*_ds_ measurements are shown for the 1T′–2H–1T′ junction at different gate voltages, ranging from 0 to 50 V. We observe a clear nonlinear behavior for the *I*_ds_ as a function of the *V*_ds_, indicative of a Schottky barrier present in our device, which could either be between the Ti/Au contact and the 1T′–MoTe_2_, or the 1T′–2H junction. To determine this, we performed the *I*_ds_–*V*_ds_ measurement on the phase-transformed 1T′ region only and observe a clear linear behavior showing Ohmic contact between the Ti/Au contacts and 1T′ region, as depicted in the inset of Figure 1c. Therefore, we expect the nonlinear behavior observed in Figure 1c to originate from a Schottky barrier between the 1T′–2H junction. Figure 1d displays the gate transfer curve of the 1T′–2H–1T′ device at *V*_ds_ = 3 V, showing a clear *n*-type transistor behavior and a threshold voltage of *V*_th_ = 40 V, confirming the semiconducting nature of the 2H phase. For the 1T′ regions, we find a two-probe resistance of 8 and 18 kΩ, which again indicate the successful transformation from the semiconducting 2H MoTe_2_ phase to the semimetallic 1T′ phase.

### Optoelectrical Characterization

To observe the optoelectronic response of the 1T′–2H–1T′ sample, we perform both scanning photocurrent measurements and time-resolved photocurrent measurements, as described in the Methods section. Unless otherwise stated, the optoelectrical measurements were performed at room temperature and high vacuum (1 × 10^–6^ mbar) conditions. First, the scanning photocurrent measurements are presented, which give more insights into the origin of the photocurrent, after which the time-resolved photocurrent measurements are discussed.

**Figure 2:**
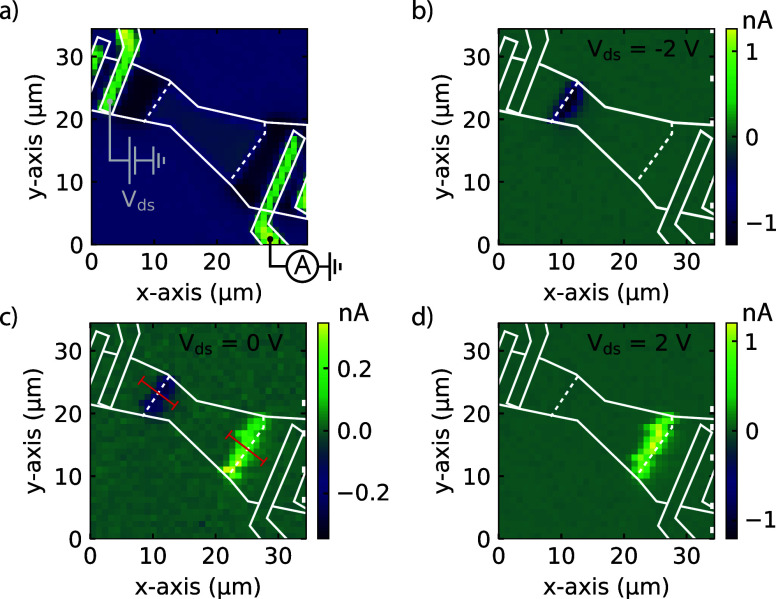
(a) Reflectivity map of the scanning photocurrent measurement of the device depicted in Figure 1a with the corresponding photocurrent map in (b), (c), and (d), taken at RT. The white outlines indicate the position of the flake and Ti/Au contacts, while the white dashed lines indicate the 1T′–2H junctions for clarity. The photocurrent maps are obtained with λ = 700 nm, *P* = 1 μW, and a *V*_ds_ of (b) −2, (c) 0, and (d) 2 V. From the photocurrent maps, we can clearly see that the induced photocurrent originates from the 1T′–2H junction rather than from the Ti/Au contacts.

The scanning photocurrent measurements enable us to spatially identify where the photocurrent is generated and thus allow us to check whether the photocurrent originates from the Ti/Au contacts or from the MoTe_2_ flake itself. When performing the scanning photocurrent measurements, the laser beam is focused and scanned across the sample in a raster-like fashion while recording both the reflection and the generated photocurrent. Figure 2a shows the recorded reflection map with an illumination wavelength of 700 nm, a power of 1 μW, and a full width at half-maximum (FHWM) spot size of 0.70 ± 0.02 μm (see Supporting Information). The contours of the flake and the Ti/Au contacts are clearly visible and highlighted with the white outlines for clarity. It should be noted that the different phases of MoTe_2_ can also be clearly distinguished, and their junctions are highlighted with the white dashed line. The corresponding photocurrent maps with a *V*_ds_ of −2, 0, and 2 V are depicted in Figure 2b–d, respectively. We clearly observe that the photocurrent originates locally from the 1T′–2H junction, rather than the Ti/Au contacts, which is the case when directly contacting the 2H MoTe_2_. This, again, confirms a low Schottky barrier between the Ti/Au and 1T′ MoTe_2_, indicating a successful phase transformation.

**Figure 3:**
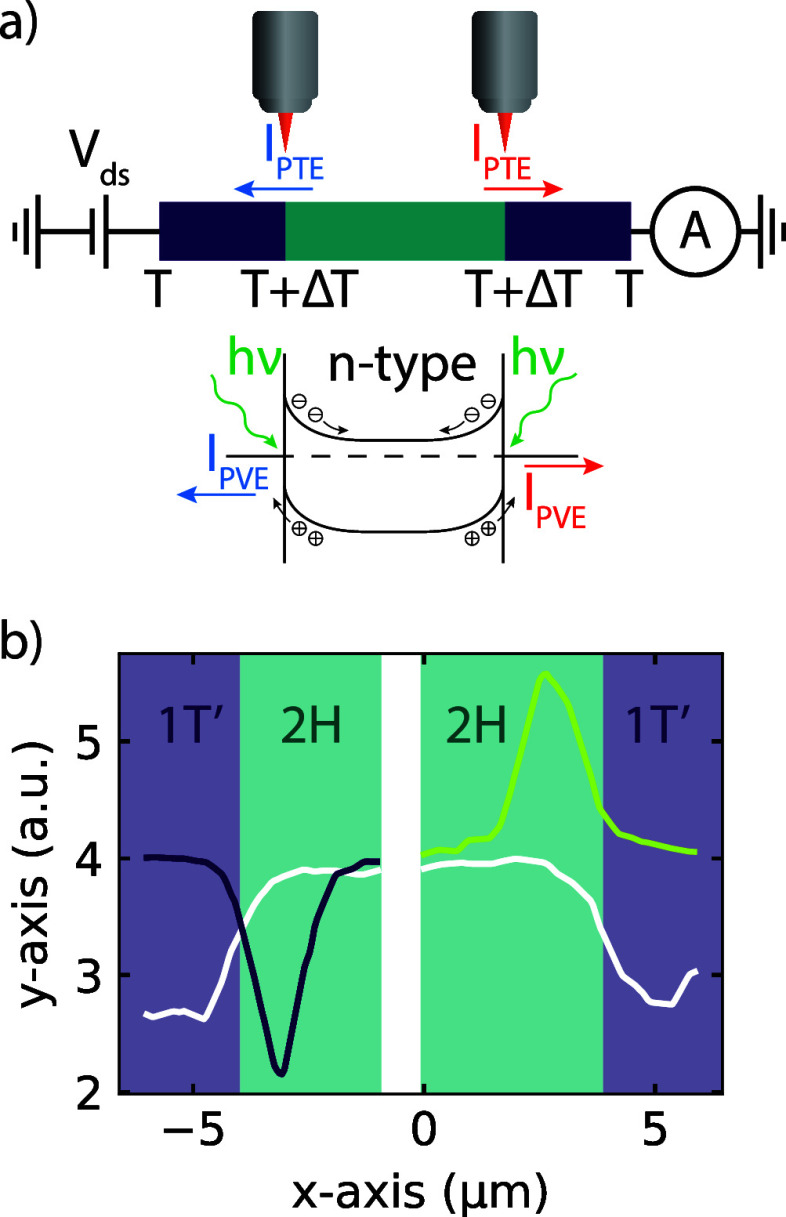
(a) Schematic of the photothermoelectric (PTE) and photovoltaic effect (PVE). For the PTE, the laser locally heats the device, which creates a temperature gradient, which via the Seebeck effect causes an induced photocurrent (*I*_PTE_). For the PVE, the localized electric field at the Schottky barrier causes a separation of the photoinduced carriers, resulting in *I*_PVE_. It should be noted that the two effects produce a photocurrent with the same direction and that the induced photocurrent is opposite on both junctions. (b) Line trace of the photocurrent mapping, as indicated in red in Figure 2c, showing the reflection (white) and negative (purple) and positive (green) photocurrent peaks along the line trace. Both the maximum and minimum photocurrents are obtained in the 2H region, as expected for a localized electric field from a Schottky barrier. The phases are indicated by the purple (1T′) and green (2H) backgrounds.

We observe photocurrents with opposite signs at the two 1T′-2H junctions for *V*_ds_ = 0 V. This is in line with the expected behavior for two possible mechanisms: photovoltaic effect (PVE) due to a Schottky barrier at the 1T′–2H junction or the photothermoelectric effect (PTE) due to a different Seebeck coefficient of the two MoTe_2_ phases. A more detailed analysis of the influence of the *V*_ds_ and the *V*_g_ on the photocurrent is shown in the Supporting Information. The two mechanisms are schematically depicted in Figure 3. While both the PVE and the PTE can give rise to photocurrent in the 2H–1T′ interface, we conclude that the PTE contribution should be negligible. For the PTE, local heating due to the laser irradiation causes a temperature gradient (Δ*T*) which is converted into a voltage difference (*V*_PTE_) due to a difference in the Seebeck coefficient between the 1T′ (*S*_1T*′*_) and 2H (*S*_2H_) phases.^9,33^ By using the maximum-generated photocurrent in our measurements, we calculate a range of unrealistic temperature gradients above 7000 K, indicating that the PTE cannot solely explain the observed photocurrent. A more detailed explanation can be found in the Supporting Information.

On the other hand, for the PVE-driven photocurrent, the localized electric field at the 1T′–2H interface causes the photoinduced electron–hole pairs to separate, resulting in a photocurrent.^33^ For our *n*-type MoTe_2_, the band alignment is depicted in Figure 3a. The electric field from the Schottky barrier is positioned in the 2H region. By taking a line scan of the reflection map and photocurrent map, indicated by the red line in Figure 2c, we can more accurately determine the position of the photocurrent peak with respect to the 1T′–2H junction. Here, we find that the peak of the photocurrent arises in the 2H region rather than at the 1T′–2H junction, which is in line with the expectation for the PVE.^32^ Additionally, we can estimate the depletion region due to the Schottky barrier by using the following equation:
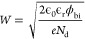
1where *W* is the width of the Schottky barrier, *e* is the electron charge, and ϵ_0_ is the permittivity of free space. By assuming a donor density *N*_d_ of 10^11^ cm^2^,^34^ a barrier height ϕ_bi_ of 60 meV,^29^ and a relative ϵ_r_ of 12, we estimate *W* to be ∼2 μm, which corresponds well to the FWHM of 1.5 ± 0.2 μm we find by fitting the positive photocurrent peak with a Gaussian.

Buscema et al. observed a similar photocurrent sign in their scanning photocurrent measurements on an *n*-type MoS_2_ photodiode.^35^ However, they attribute the induced photocurrent to the PTE, as they observe clear photocurrent generation in the center of their Ti/Au contacts and see a linear *I*_ds_–*V*_ds_ behavior with no indication of a Schottky barrier. In contrast, for our 1T′-2H MoTe_2_ junctions, we observe a clear nonlinear IV-curve, indicating that the Schottky barrier plays a more important role in our devices. Furthermore, they see a pronounced photocurrent even when exciting below the bandgap of MoS_2_. Unfortunately, our optoelectronic setup only allows for excitation up to 1100 nm, which is still above the bandgap of MoTe_2_ (∼1.1 eV ∝ ∼1127 nm for bulk).^36,37^ Therefore, we suggest further research to be performed on below-band gap excitation to determine to what extent the PTE is contributing to the observed photocurrent.

**Figure 4:**
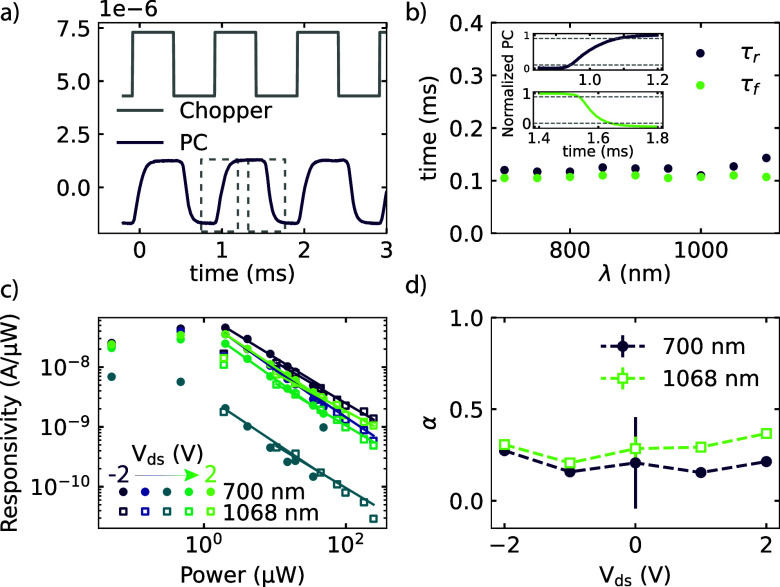
(a) Time-resolved photocurrent, taken at RT, where the photocurrent (purple) in the device is plotted together with the chopper signal (gray) versus time, shows the fast response of our MoTe_2_ photodetector. The dashed gray rectangles indicate the region used to determine the rise and fall times, as depicted in the inset of (b). (b) Extracted rise (purple) and fall times (green) indicate no wavelength dependence on the fast response for wavelengths ranging from 700 to 1100 nm. The inset shows the rise (purple) and fall (green) curves of the photocurrent from which the rise and fall times are extracted. (c) Power-dependent measurements for different *V*_ds_, ranging from −2 to 2 V, with a maximum responsivity of 4.5 × 10^–8^ A/μW. Here, the responsivity (*R*) of the device is plotted as a function of the laser excitation power (*P*) and fitted at high laser excitation power to a power law: *R* ∝ *P*^α–1^. The measured *R* for 700 and 1068 nm are indicated by the filled circles and unfilled squares, respectively. (d) Extracted index of the power law (α) from the fitting in (c) versus *V*_ds_ for the wavelengths 700 nm (purple) and 1068 nm (green).

To characterize the optoelectronic performance of our MoTe_2_ photodetector, we perform time-resolved and power-dependent photocurrent measurements, as depicted in Figure 4. By measuring the induced photocurrent versus time, using a chopper to modulate the light on and off (see Figure 4a), we are able to extract the rise (τ_r_) and fall times (τ_f_) of the device, which are defined as the time required for the photocurrent to increase from 10 to 90%, and decrease from 90 to 10% of the maximum photocurrent, respectively. By performing these measurements over a range of different excitation wavelengths, we find that we get short rise and fall times of ∼113 and ∼110 μs, respectively, independent of the wavelength, as depicted in Figure 4b. These response times correspond to a 3 dB frequency of 0.35/τ_r_ = 3 kHz,^38^ which are close to the performance of graphene/MoTe_2_/graphene photodetectors.^39^ In contrast, when directly contacting the 2H MoTe_2_ with Ti/Au electrodes, we find a much slower response (over 100-fold slower), as shown in Figure S2c in the Supporting Information. Here, we observe a waveform similar to a “*sawtooth*” response, indicative of capacitive behavior, at 20 Hz. This is a result of the highly resistive contact with the 2H region of the device, leading to photogating and slow charging of the device. This shows that using the 1T′ regions to contact the 2H MoTe_2_ increases the temporal response of our MoTe_2_ photodetector by more than 2 orders of magnitude.

The response dynamics displayed at the 1T′–2H junction are fast compared to other TMD-based photodetectors.^38,40^ More specifically, compared to other MoTe_2_-based photodetectors, they are 1 order of magnitude faster than the report of Huang et al. on directly contacted 2H MoTe_2_,^41^ and similar to the ones found by Lin et al. in 1T′–contacted 2H MoTe_2_.^29^ On other TMD-based devices, a variety of different response dynamics are reported, with the fastest responses reported for deep UV and mid-IR detectors on graphene/MoTe_2_/black phosphorus devices, which reach bandwidths of 2.1 MHz.^42^

To determine the responsivity of our devices, we vary the excitation power at a fixed excitation wavelength (700 and 1068 nm). From these power-dependent measurements, we are able to determine the responsivity by *R* = *I*_PC_/*P*, where *I*_PC_ is the induced photocurrent, and *P* is the power of the laser,^9,33^ and find a maximum *R* of 4.5 × 10^–8^ A/μW with a wavelength of 700 nm and a 2 V bias. The value we find is comparable to other reports on TMD-based photodetectors, ranging approximately from 7.25 × 10^–11^ A/μW to 9.708 × 10^–3^ A/μW.^40,41,43,44^ For the same wavelength and bias voltage, we also calculate a maximum external quantum efficiency (EQE) of ∼8%. Given that there is commonly a trade-off between fast response and high responsivity in these devices,^38^ it is not unexpected that both the EQE and the responsivity of our devices fall within the middle or lower half of reported values.^30,39,45^ Additionally, the responsivity of our device is measured with a focused laser spot rather than illuminating the entire device. This could lead to an underestimation of the generated photocurrent, as only a small fraction of the photodetector area is used to generate the photocurrent. Moreover, the capacitance added by the global back-gate can lead to an increase in the response times of our device.

Figure 4c clearly shows a decrease of responsivity with incident excitation power for *P* > 1.9 μW, which is commonly observed in TMD photodetectors.^46,47^ It can be associated with a reduced number of photogenerated carriers available for extraction under high photon flux due to the saturation of recombination/trap states that influence the lifetime of the generated carriers.^48^ The responsivity versus laser power can be expressed by a power law *R* ∝ *P*_d_^α–1^ for *P* > 1.9 μW, where *P* is the laser power, and α is the index of the power law.^41,49^ From the fit, we are able to extract α for the two different wavelengths at different *V*_ds_ values, as shown in Figure 4d. The deviation from the ideal slope of α = 1, where the responsivity does not depend on the laser power, can be attributed to complex processes in the carrier generation, trapping, and electron–hole recombination in MoTe_2_.^50,51^ For the PTE, a value of α ∼ 0.8 is expected, while we find a value of α ∼ 0.25, which indicates again that the PTE is not primarily responsible for the generated photocurrent in our device.^31^

## Conclusions

In conclusion, laser-induced phase transformation is a simple, scalable, and reliable methodology to engineer MoTe_2_ optoelectronic devices. Our results indicate that contacting the 2H region of MoTe_2_ via a phase-transformed 1T′ region is beneficial for the temporal optoelectronic response of MoTe_2_-based photodetectors and does not require complex heterostructure fabrication. Our scanning photocurrent measurements and nonlinear IV curves clearly show that the origin of the photocurrent in our devices can be ascribed to the Schottky barrier between the 1T′ and 2H junction, rather than the photothermoelectric effect or Schottky barriers at the Ti/Au electrode-TMD interface. Contacting MoTe_2_ via the phase-transformed 1T′ region, therefore, allows one to study the intrinsic properties of the TMD rather than the electrode-TMD interactions, beneficial for fundamental research. Additionally, an increase of 2 orders of magnitude in the optoelectronic temporal response is observed when contacting the 2H MoTe_2_ via the 1T′ regions. This shows that tailoring the crystallographic phase of TMDs locally and altering their optoelectronic response at will can have a profitable effect on the optoelectronic operation. Our results, in combination with the wide variety of phase-engineering techniques and different TMDs available, could lead to a further improved performance of TMD-based optoelectronic devices, leading to more sensitive, faster, and flexible photodetectors.

## Methods

### Device Fabrication

The 2H MoTe_2_ flakes are obtained by mechanical exfoliation (bulk crystal supplied by HQ graphene) and transferred onto a Si/SiO_2_ (285 nm) substrate in a nitrogen environment. Using an optical microscope, the MoTe_2_ flakes are selected based on their size, thickness, and homogeneous surface. Details of the selection criteria, as well as Figure S4, a discussion on the impact of the flake thickness to the laser-induced phase change, can be found in the Supporting Information. Next, the Raman spectra are obtained with an inVia Raman Renishaw microscope using a linearly polarized laser in backscattering geometry. The excitation wavelength and grating used were λ = 532 nm and 2400 l/mm, respectively. The laser power was ∼100 μW with a diffraction-limited spot of ∼1 μm. Using the same system, the 2H–1T′ phase transformation is performed by selectively illuminating parts of the MoTe_2_ flake with the 532 nm laser beam in a raster-like fashion, using steps of 500 nm and 0.1 s illumination. We find that a laser power of ≥3.25 mW (laser spot size around 500 nm) is needed to initiate the phase transformation. Finally, using standard lithography techniques, the Ti/Au (5/55 nm) contacts are fabricated on top of the flake by means of electron beam lithography and electron beam evaporation.

### Optoelectronic Measurements

The electrical characterization (i.e., IV-sweeps, transfer curves) is performed using Keithleys 2400 and 2450 source measure units at 78 K. For the optoelectronic measurements, a supercontinuum white light laser (NKT Photonics SuperK EXTREME) is used as the illumination source, and the measurements are taken at room temperature. The induced photocurrent is measured in a short-circuit configuration using a Stanford Research Systems SR830 lock-in amplifier, which is referenced to the frequency of the optical chopper. The photocurrents are either measured directly by the lock-in amplifier or converted to a voltage using a home build current preamplifier, which is subsequently measured by the lock-in amplifier. The time-resolved photoresponse of the device, as depicted in Figure 3a, is measured using a chopper and an oscilloscope (Keysight DSOX1204A) at room temperature.

## Supporting Information Available

Details over the photothermoelectric temperature gradient calculation; laser spot size calculation and data for 700 and 1064 nm for the reported measurements; equation and additional details for charge carrier mobility and further details of another phase-engineered device, including responsivity and transfer curves; drain-source and gate voltage dependence of the photocurrent; normalized Raman spectra and spectral weight contribution of the 1T′ phase characteristic peaks; laser irradiation power; and flake thickness dependence for phase change (PDF)

## Related Paper

The author’s first manuscript^52^ of this publication was published, containing a preliminary version of this paper.
