# Supplementary material for: Locally Phase-Engineered MoTe2 for Near-Infrared Photodetectors
Source: ACS Photonics. 2024 Sep 16;11(10):4083–9. doi: 10.1021/acsphotonics.4c00896 (PMC11487713; doi:10.1021/acsphotonics.4c00896)
Supplement: Supplementary file 1 — ph4c00896_si_001.pdf [file ph4c00896_si_001.pdf]

Supporting Information: Locally Phase-Engineered $MoTe_2$ for Near-Infrared Photodetectors

Jan Hidding,[†,§] Cédric A. Cordero-Silis,[†,§] Daniel Vaquero,[‡] Konstantinos P. Rompotis,[†] Jorge Quereda,[¶] and Marcos H. D. Guimarães[*,†]

†*Zernike Institute for Advanced Materials, University of Groningen, 9747 AG Groningen, The Netherlands*

‡*Nanotechnology Group, USAL—Nanolab, Universidad de Salamanca, E-37008 Salamanca, Spain*

¶*Departamento de Física de Materiales, GISC, Universidad Complutense de Madrid, E-28040 Madrid, Spain*

§*These authors contributed equally*

E-mail: m.h.guimaraes@rug.nl

PTE temperature gradient calculation

In a short-circuit configuration, laser irradiation causes a temperature gradient that can lead to an induced photocurrent driven by the PTE. By using the Seebeck coefficient of the 2H and 1T' phases which were shown to be, respectively, $S_{2H} \sim 230$ µV K^{-1} and $S_{1T'} \sim 30$ µV K^{-1},[S1] we can calculate an approximate temperature gradient for our devices using the following equation:

$$V_{PTE} = (S_{1\mathrm{T}'} - S_{2\mathrm{H}})\Delta T. \quad (1)$$

Assuming that the maximum photocurrent solely originates from the PTE, the local temperature gradient can be calculated by considering the photocurrent generated at the 2H-1T' interfaces in Fig. 2(c), and the 2-probe resistance through the channel. Together with the values for the Seebeck coefficient mentioned above, we find unrealistically high temperature gradients of between 7490 K and 11952 K. This is indicative that the PTE is not the sole mechanism responsible for the generated photocurrent.

Laser spot determination

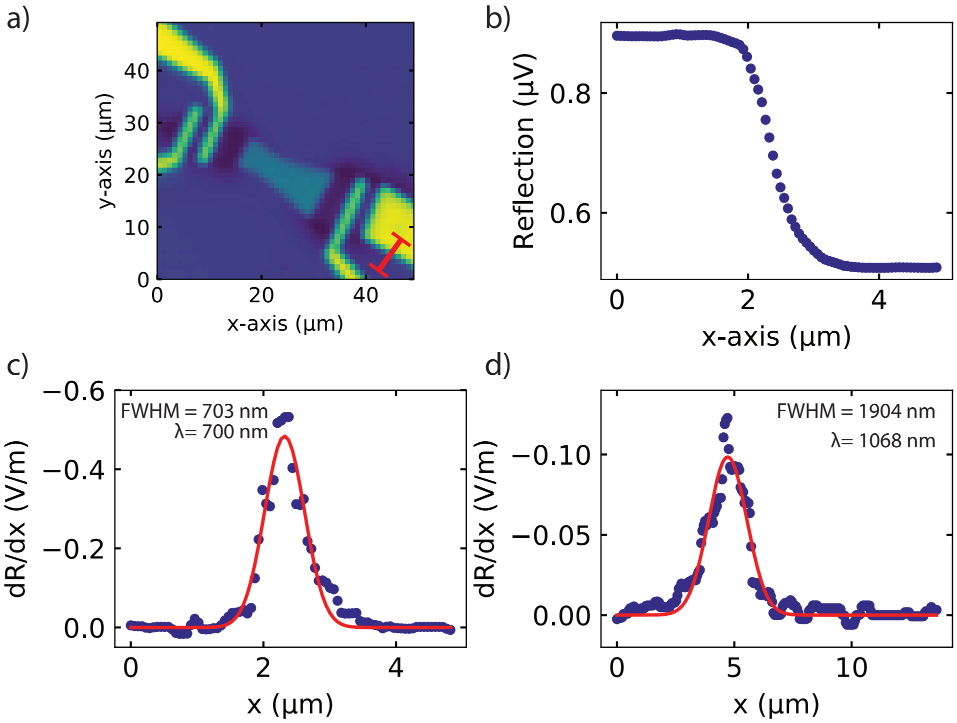

Figure S1: (a) Reflection map taken with λ = 700 nm of the device depicted in Fig. 1(a), where the red line indicated the position of the line trace to determine the laser spot size. (b) The data (blue circles) of the line trace in (a), which shows the clear step from the Ti/Au electrode to the SiO_2 substrate. (c) By taking the derivative of the data depicted in (b) (blue circles), and fitting it with a Gaussian (red line), the FWHM of the laser spot used to do the scanning is determined. (d) Same as (c) but now for λ = 1068 nm.

To determine the spot size of our laser at the wavelengths of 700 nm and 1068 nm, we use the software Gwyddion to take a line trace over a scanning map of the reflectivity. The reflectivity map for 700 nm is depicted in Fig. S1(a), where the position of the line trace is indicated with a red line and the line trace itself is depicted in Fig. S1(b). By fitting the derivative of the data in Fig. S1(b) with a Gaussian, we are able to determine the FWHM of the laser spot, as show in Fig. S1(c) and (d) for 700 nm and 1068 nm, respectively. For a wavelength of 700 nm, we find a diffraction limited FWHM of 0.70 ± 0.02 µm, while for the 1068 nm, we find a broader FWHM of 1.91 ± 0.04 µm.

Mobility

The transfer curve of Fig. 1(d), for the device depicted in Fig. 1(a), shows clear n-type behavior with a threshold voltage of $V_{th} = 40.3$ V. The mobility μ of the device is determined by:

$$\mu = \left(\frac{dI_{ds}}{dV_g}\right)\left(\frac{l}{wC_gV_{ds}}\right), \tag{2}$$

where (dI_{ds}/V_g) is the slope at positive V_g, l and w are the length and width of the channel, C_g is the area capacitance of the SiO_2 back gate (1.2×10^{-4} F/m^2), and V_{ds} is the drain-source voltage (3 V). We calculate a mobility of 0.08 cm^2/(V $\cdot$ s).

Other Phase-Engineered $MoTe_2$ Devices

We measured other phase transformed $MoTe_2$ devices, one of them exhibits clear *p*-type behavior, and the other one *n*-type. For the *p*-type device, different mobilities compared to the device depicted in Fig. 1(a) are obtained. The difference is attributed to the following explanation: When contacting the 2H $MoTe_2$ directly with the Ti/Au contacts, we find a mobility of 1.74 $cm^2/V \cdot s$, while if the 2H region is contacted via the 1T' region, similar to Fig. 1(d), we find a mobility of 14.18 $cm^2/V \cdot s$, in agreement with the reports of Bae et al.[S2] on electrical measurements on phase changed $MoTe_2$ devices and larger compared to similar measurements reported by Zhang et al.[S3]

Finally, the *n*-type phase changed $MoTe_2$ device, with the same contact geometry as the device in Figure 1(d), displays a relatively high photoresponse that, despite being one order of magnitude lower to the device in Figure 1(d), is still comparable to previously reported devices.[S4] As in the case for Figure 4(a), the calculated rise and fall times of this device are, respectively, $\tau_r = 1.26$ ms and $\tau_f = 1.12$ ms, with a 3 dB frequency of 0.277kHz.

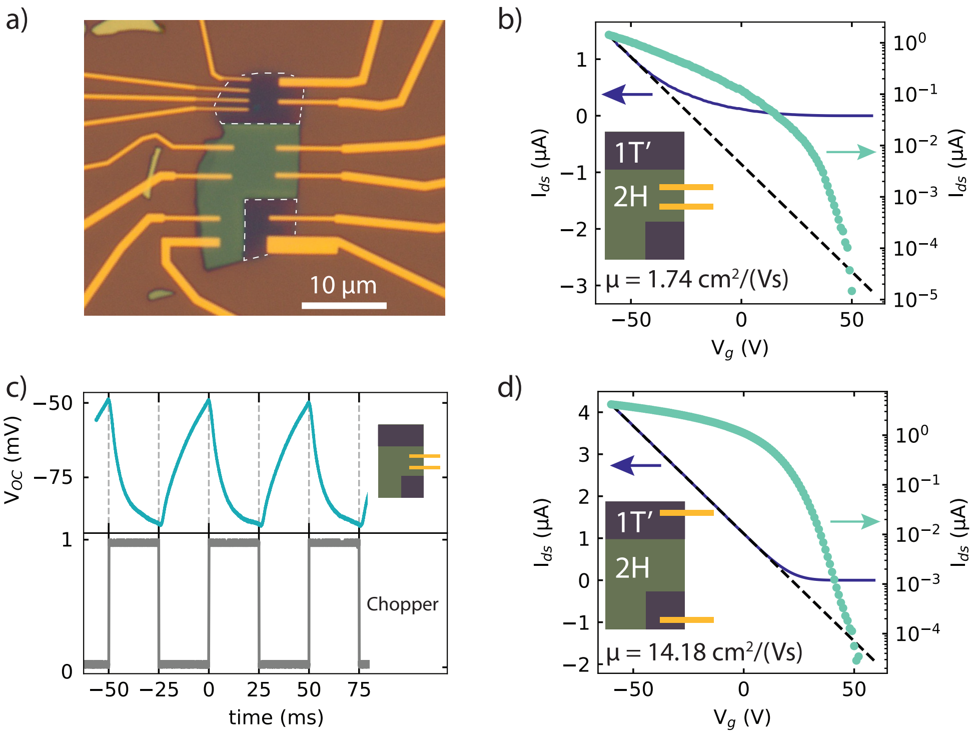

Figure S2: (a) Optical micrograph of another phase-engineered $MoTe_2$ device similar to the device depicted in Fig. 1(a) in the main text. The white dashed regions indicate the areas where the 2H $MoTe_2$ are transformed to 1T' by laser irradiation. (b) Transfer curve measured with the Ti/Au electrodes directly deposited on the 2H region, which shows clear p-type behavior. (d) The transfer curve measured with the Ti/Au electrodes on the 1T' region. By fitting the curve on negative gate voltages and using Eq. 2, we are able to extract a mobility of 1.74 $cm^2/(V \cdot s)$ and 14.2 $cm^2/(V \cdot s)$, respectively. (c) The top panel shows the temporal photovoltage response (green) of the $MoTe_2$ device when the 2H crystal is directly contacted with the Ti/Au electrodes. The bottom panel depicts the signal from the chopper (grey), which chops the light on and off. A much slower optoelectronic response is observed compared to the response of the device discussed in the main text, as shown in Fig. 4(a).

Drain-source voltage dependence of the photocurrent

In order to give a more comprehensive understanding of the photocurrent generation in the 1T'-2H-1T' $MoTe_2$ devices, we characterize the dependence of I_{pc} with the voltage bias, V_{ds}. As shown in the main text, I_{pc} is generated locally in the 1T'-2H junctions because the local electric field induces the separation of the photogenerated electron-hole pairs. Figure S3 shows the I_{pc} generated at the 2H-1T' and 1T'-2H junctions as a function of V_{ds} for gate voltage values of $V_g = 0$ V, 20 V and 40 V. These measurements were performed at 78K to reduce the gate leakage current at higher gate voltages. Figure S3a depicts the case where the 2H-1T', electrically grounded, junction is illuminated. In this scenario, for negative values of V_{ds}, we observe a negligible photocurrent. Conversely, when $V_{ds} > 0$ V, I_{pc} increases. The inset illustrates the band diagrams for both negative and positive V_{ds}. For $V_{ds} > 0$ V the localized electric field at the 2H-1T' interface increases the amount of photogenerated holes that reach the 1T' region, resulting in an increase of the measured I_{pc}. In contrast, when $V_{ds} < 0$, I_{pc} decreases because the Schottky barrier hinders the electron flow to the 1T' region. Figure S3b shows the I_{pc} generated near the 1T'-2H junction on the other side of the device, which is biased. Here we have the opposite behaviour, for negative values of V_{ds}, we observe a negative I_{pc}, while when $V_{ds} > 0$ V, I_{pc} diminishes. The band diagrams in the inset show that when $V_{ds} < 0$ V the photogenerated holes reach the 1T' contact. However, with a positive V_{ds}, I_{pc} diminishes as the Schottky barrier prevents a high electron flow across the 2H-1T' junction.

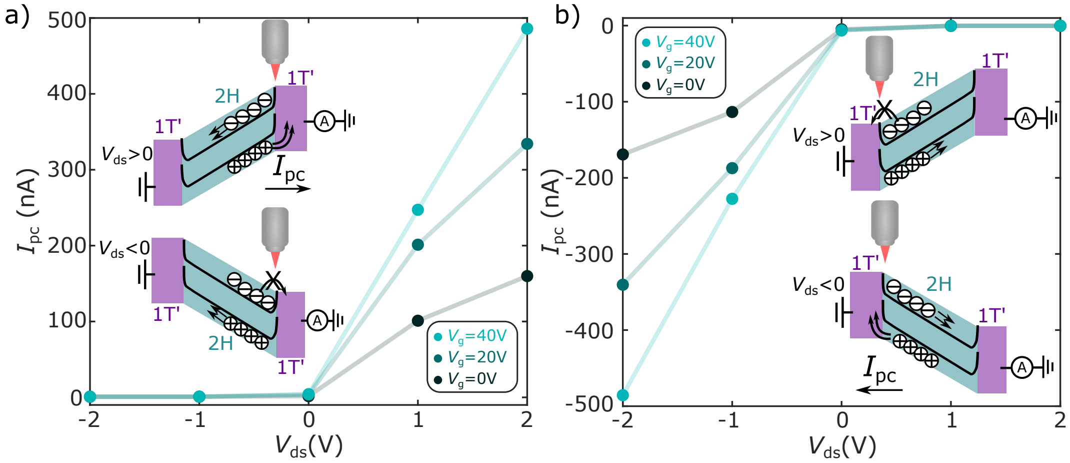

Figure S3: I_{pc} generated near the 2H-1T' (a) and 1T'-2H (b) junctions of the device shown in the main text. I_{pc} is depicted as a function of V_{ds}, measured at various values of V_g. The insets illustrate the band diagrams of the device under negative and positive bias conditions, highlighting the I_{pc} generated in each scenario. These measurements were performed at 78 K.

Gate voltage dependence of the photocurrent

Figure S4 shows the dependence of I_{pc} on V_g for two $MoTe_2$ phase-engineered devices: one showing n-type behavior (a and b) and the other p-type (c and d). Measurements for both devices were performed at 78K. It is evident for both devices that as V_g is brought above the threshold voltage, *i.e.* more positive V_g for the n-type device and more negative V_g for the p-type device, the photocurrent also rises. Both devices exhibit behavior similar to other TMD-based devices, where the gate-induced increase in charge carrier density leads to a rise in photocurrent.[S5,S6]

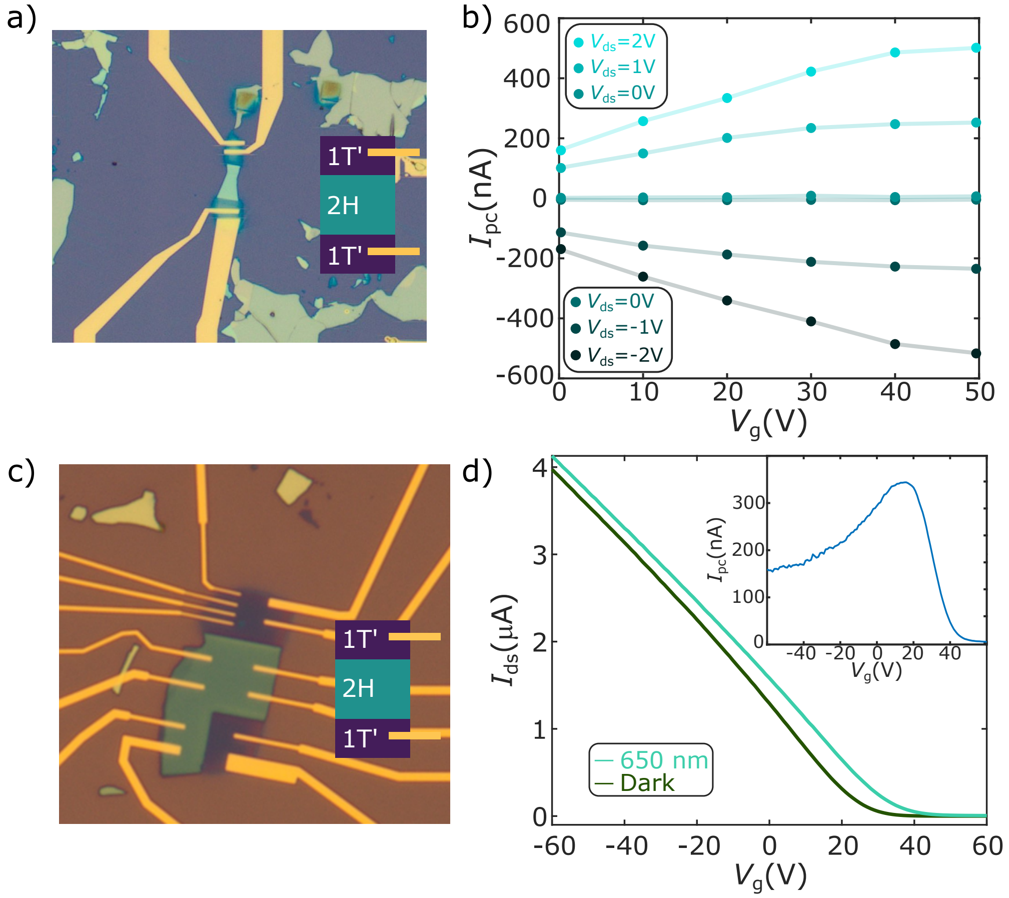

Figure S4: Gate voltage dependence of the photocurrent. Optical image of a n-type device (a) and its respective gate voltage dependence of the photocurrent measured at different values of the drain source voltage (b). The illumination wavelength in these measurements is 700 nm. Optical image of the phase-engineered $MoTe_2$ p-type device (c) and its gate transfer curves (d) under an illumination of 650 nm light and dark conditions. The inset shows the dependence of the photocurrent with the gate voltage. All measurements here were performed at 78 K.

Additional Raman Characterization

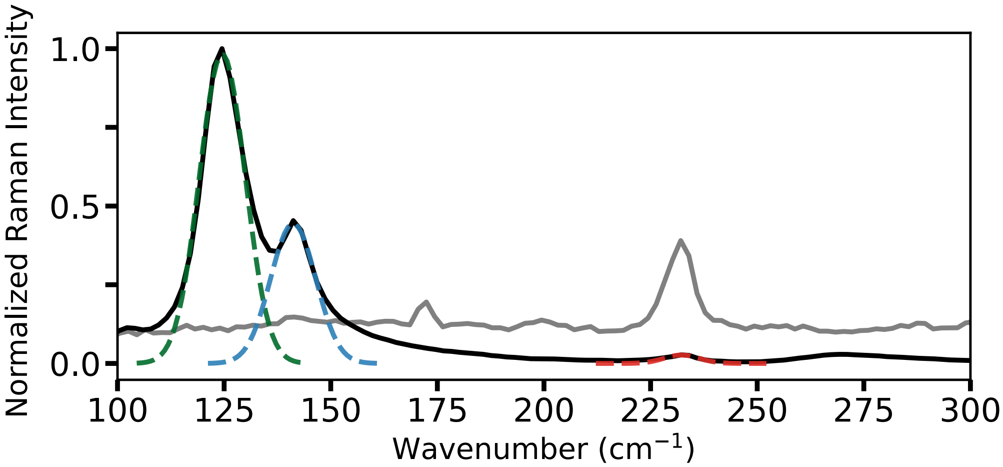

Figure S5: Normalized Raman spectra of a phase changed 1T' area (in black) and 2H phase (in grey). Gaussian fits are done to each peak in order to calculate the contribution to each peak to the 1T' Raman spectra.

Table S1: Extracted parameters (center, FWHM, amplitude, integrated area and spectral weights) from the Raman spectra of the 1T' phase in Figure S5.

**Fitted peak center (cm^{-1})**	**FWHM (cm^{-1})**	**Amplitude**	**Area**	**Spectral Weight**
124.58	12.65	0.982	13.23	2.14
141.19	12.49	0.444	5.91	0.44
232.28	9.76	0.026	0.28	0.01

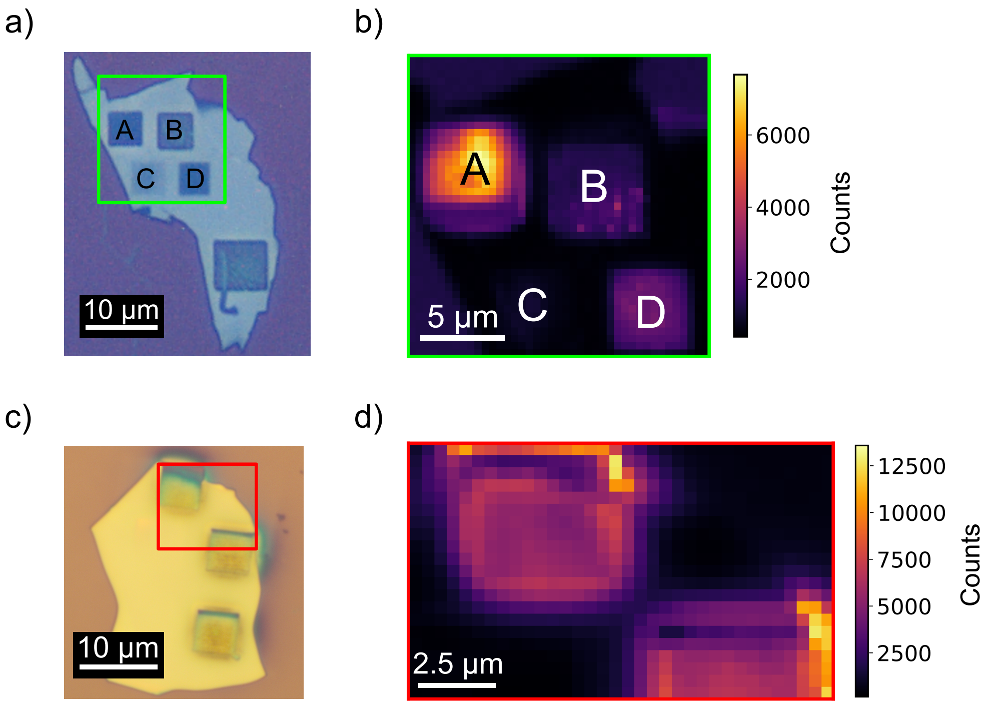

Figure S6: Effect of the laser irradiation power and $MoTe_2$ thickness on the phase change. (a) Optical micrograph of a $MoTe_2$ flake irradiated with different laser powers: A = 9.2 mW, B = 16.3 mW, C and D = 2.1 mW irradiated 10 and 12 times respectively. (b) Raman intensity for the region highlighted in (a) for the 1T' A_g peak at 124 cm^{-1}. (c) Optical micrograph of a thicker $MoTe_2$ flake irradiated using the same laser threshold power obtained in region A of panel (a) - 9.2 mW. (d) Corresponding Raman mapping for the 1T'A_g peak at 124 cm^{-1} in the region highlighted in (c).

In order to determine the optimum laser irradiation conditions for a range of $MoTe_2$ flake thicknesses we perform different irradiation experiments in several flakes. Figure S6a displays a 2H-$MoTe_2$ flake with similar thickness as the ones used for our devices, around 25 nm, where we perform phase change experiments using different laser powers. The higher power laser (region B) induces the phase change but with an increased flake thinning - *i.e.* the laser power is well beyond the necessary threshold. Consecutive scans at lower power can induce a partial phase change (as seen in regions C and D), while the best result is at 9.2 mW. Figure S6c shows a thicker flake ($\sim$ 80 nm) where the phase change process used was at the same threshold power obtained for the thinner flake (9.2 mW), resulting in a homogenous complete phase change as displayed in the Raman mapping (Fig. S6d) of the 1T' A_g band.

References

(S1) Keum, D. H.; Cho, S.; Kim, J. H.; Choe, D.-H.; Sung, H.-J.; Kan, M.; Kang, H.; Hwang, J.-Y.; Kim, S. W.; Yang, H.; Chang, K. J.; Lee, Y. H. Bandgap opening in few-layered monoclinic $MoTe_2$. Nature Physics **2015**, 11, 482–486.

(S2) Bae, G. Y.; Kim, J.; Kim, J.; Lee, S.; Lee, E. $MoTe_2$ Field-Effect Transistors with Low Contact Resistance through Phase Tuning by Laser Irradiation. Nanomaterials **2021**, 11, 2805.

(S3) Zhang, X.; Jin, Z.; Wang, L.; Hachtel, J. A.; Villarreal, E.; Wang, Z.; Ha, T.; Nakanishi, Y.; Tiwary, C. S.; Lai, J.; Dong, L.; Yang, J.; Vajtai, R.; Ringe, E.; Idrobo, J. C.; Yakobson, B. I.; Lou, J.; Gambin, V.; Koltun, R.; Ajayan, P. M. Low Contact Barrier in 2H/1T' $MoTe_2$ In-Plane Heterostructure Synthesized by Chemical Vapor Deposition. ACS Applied Materials and Interfaces **2019**, 11, 12777–12785.

(S4) Huang, H.; Wang, J.; Hu, W.; Liao, L.; Wang, P.; Wang, X.; Gong, F.; Chen, Y.; Wu, G.; Luo, W.; Shen, H.; Lin, T.; Sun, J.; Meng, X.; Chen, X.; Chu, J. Highly Sensitive Visible to Infrared $MoTe_2$ Photodetectors Enhanced by the Photogating Effect. Nanotechnology **2016**, 27, 445201.

(S5) Island, J. O.; Blanter, S. I.; Buscema, M.; van der Zant, H. S. J.; Castellanos-Gomez, A. Gate Controlled Photocurrent Generation Mechanisms in High-Gain In_2Se_3 Phototransistors. Nano Letters **2015**, 15, 7853–7858.

(S6) Vaquero, D.; Clericò, V.; Salvador-Sánchez, J.; Díaz, E.; Domínguez-Adame, F.; Chico, L.; Meziani, Y. M.; Diez, E.; Quereda, J. Fast response photogating in monolayer MoS_2 phototransistors. Nanoscale **2021**, 13, 16156–16163.
